# An aging, pathology burden, and glial senescence build-up hypothesis for late onset Alzheimer’s disease

**DOI:** 10.1038/s41467-023-37304-3

**Published:** 2023-03-25

**Authors:** Victor Lau, Leanne Ramer, Marie-Ève Tremblay

**Affiliations:** 1 Division of Medical Sciences, University of Victoria, Victoria, BC Canada; 2 Institute on Aging and Lifelong Health, University of Victoria, Victoria, BC Canada; 3 Centre for Advanced Materials and Related Technology (CAMTEC), University of Victoria, Victoria, BC Canada; 4 Department of Biomedical Physiology & Kinesiology, Simon Fraser University, Burnaby, BC Canada; 5 Axe Neurosciences, Centre de recherche du CHU de Québec-Université Laval, Québec, QC Canada; 6 Département de Médecine Moléculaire, Faculté de Médecine, Université Laval, Québec, QC Canada; 7 The Department of Biochemistry and Molecular Biology, The University of British Columbia, Vancouver, BC Canada; 8 Department of Neurology and Neurosurgery, McGill University, Montréal, QC Canada

**Keywords:** Alzheimer's disease, Cognitive ageing, Microglia, Neuroimmunology, Complexity

## Abstract

Alzheimer’s disease (AD) predominantly occurs as a late onset (LOAD) form involving neurodegeneration and cognitive decline with progressive memory loss. Risk factors that include aging promote accumulation of AD pathologies, such as amyloid-beta and tau aggregates, as well as inflammation and oxidative stress. Homeostatic glial states regulate and suppress pathology buildup; inflammatory states exacerbate pathology by releasing pro-inflammatory cytokines. Multiple stresses likely induce glial senescence, which could decrease supportive functions and reinforce inflammation. In this perspective, we hypothesize that aging first drives AD pathology burden, whereafter AD pathology putatively induces glial senescence in LOAD. We hypothesize that increasing glial senescence, particularly local senescent microglia accumulation, sustains and drives perpetuating buildup and spread of AD pathologies, glial aging, and further senescence. We predict that increasing glial senescence, particularly local senescent microglia accumulation, also transitions individuals from healthy cognition into mild cognitive impairment and LOAD diagnosis. These pathophysiological underpinnings may centrally contribute to LOAD onset, but require further mechanistic investigation.

## Abstract (web summary)

In this perspective, the authors hypothesise that glial senescence, requiring senescent microglia burden, perpetuates further aging, Alzheimer’s pathologies, and senescence. Increasing glial senescence is proposed as necessary to drive individuals from healthy cognition into cognitive decline and dementia.

## Introduction

Alzheimer’s disease (AD) is characterized by cognitive decline, most prominently manifested as progressive memory loss. AD risk factors include vascular pathology, declining metabolism, and most significantly, aging^1–4^. These risk factors contribute towards sporadic or late onset AD (LOAD) (>90% of AD cases), and early-onset or inherited, familial AD (EOAD)^3^. In LOAD, synaptic loss is pathologically correlated with neurodegeneration and cognitive decline^1,2^. Widely accepted explanations for neuronal loss and LOAD progression have been proposed in the tau hypothesis and amyloid cascade (Box 1). While clinical trials targeting these two pathologies will hopefully halt AD^3,4^, focuses on understanding AD have more recently expanded into next-generation sequencing, aging-related oxidative stress, and glia. Particularly, genetic risk factors such as *TREM2* and *APOE* have exclusive roles in glia among the central nervous system^3^. Some of these genes mediate increased phagocytic function in glia, whereas others allot glia towards adopting pro-inflammatory states leading to chronic damage to cell machinery (i.e., lipids, proteins, nucleic acids) and premature cell death. Possible roles of glial senescence in AD were also recently explored^5,6^.

Cellular senescence is an irreversible cell arrest caused by replicative senescence or local, environmental challenges^7–9^. Senescence induction and characterization is heterogeneous, varying across cell types, as well as physiological and pathological environments; DNA damage, oxidative stress via the p38 MAPK pathway, and the NLRP3 inflammasome can induce senescence^9–11^. Due to heterogeneity, no universal markers for senescence are currently available^7^. Senescent cells gain apoptotic resistance with organelle dysfunction, increased levels of reactive oxygen species, and substrate build-up^7^. These substrates can comprise senescence-associated beta-galactosidase (SA-β-gal), ferritin, iron, and lipofuscin accumulation^7,12,13^. Other senescence markers include p16^INK4A^ and p21 upregulation relating to cell cycle arrest, and senescence-associated heterochromatin foci formation^7^. Senescent cells often adopt a senescence-associated secretory phenotype (SASP) involving cGAS-cGAMP-STING signaling, subsequent release of type I interferons (IFN), matrix metalloproteases (MMPs), HMGB1 protein, and other mediators varying across cell types^7,11,14^. SASP regulators include NF-κβ, C/EBP, and HMGB1^9,10^.

Senescence is considered to play physiological roles, yet with aging it becomes implicated with accelerated mortality^7^. Senolytic drugs selectively removing senescent cells increase lifespan and quality of life in mice^15–17^. Although senescence involvement in LOAD has been explored^5,6,18–20^, how senescence relates to LOAD and Braak staging is not well defined. We hypothesize that LOAD progresses based on the (i) severity of glial cell aging and (ii) maladaptive senescence accumulation corresponding to Braak staging. This perspective presents this hypothesis by first explaining LOAD and aging, followed by discussing evidence of glial dysfunction and decline in LOAD. We then propose integrated mechanisms involving AD pathology, glial senescence induction, and neurodegeneration. We predict that an accumulation of senescent microglia in local brain regions may (i) enhance Aβ and hp-tau aggregate spread, (ii) enhance synaptic loss, and (iii) induce paracrine senescence in nearby cells, resulting in heterogeneous, local spread converging into Braak staging progression.

Box 1: A brief summary of Braak staging and dominant LOAD theories of pathology The Amyloid Cascade and Tau hypotheses are the best characterized mechanisms for explaining synaptic loss and neurodegeneration observed over LOAD progression.

**Fig. 1:**
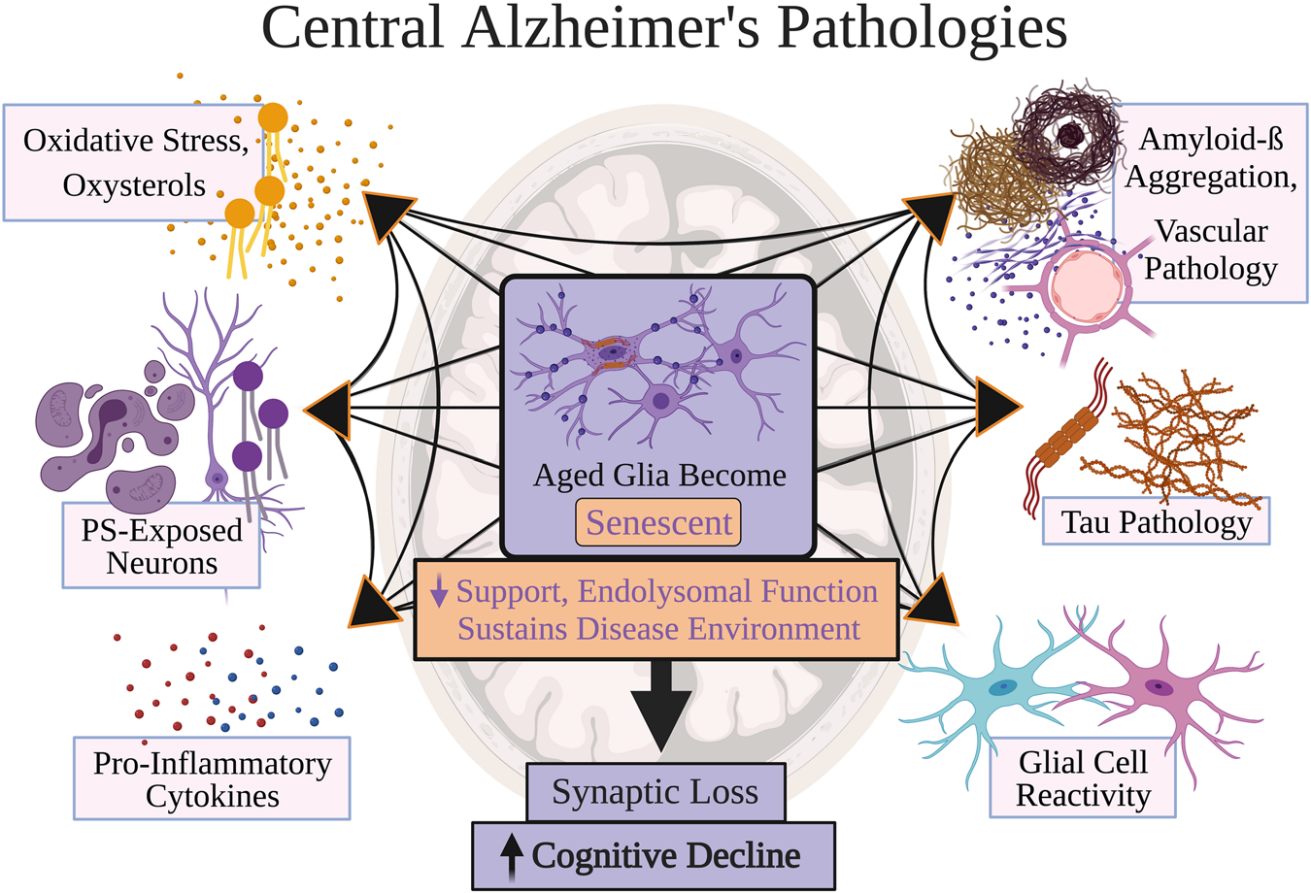
Central Alzheimer’s pathologies. Risk factors for late-onset Alzheimer’s disease (LOAD) are proposed to contribute to at least one of six main pathologies: oxidative stress and oxysterol production, phosphatidylserine (PS)-exposed neurons, pro-inflammatory cytokines, amyloid-beta aggregation and vascular pathology, tau pathology, and glial cell reactivity. Each main pathology likely eventually adds to and increases the burden of other main AD pathologies. These pathologies may ultimately converge to drive improper neuronal support, synaptic loss, and death, resulting in neurodegeneration clinically corresponding to cognitive decline. Centrally, the repeated buildup of these six LOAD pathologies proposedly pushes an aged glial system into senescence induction and accumulation. This burden of senescent glia is predicted to sustain a disease environment that determines local neurodegeneration involved in LOAD progression. Glial senescence is briefly characterized as dysfunctional glial states with impaired endolysosomal function and support for nearby cells. What constitutes glial senescence burden may well vary per an individual’s genetics, physiology, and environmental experiences; nonetheless, it is predicted that the glial senescence burden differentiates a cognitively healthy or resilient individual from one diagnosed with LOAD. Purple color designates associations with senescence and dementia progression. Figure created with BioRender.com.

The *Amyloid Cascade Hypothesis* asserts that amyloid-beta (Aβ) protein accumulation, especially Aβ oligomers, acts through multiple pathways to cause neurodegeneration and synaptic loss^2,3^. Misfolded Aβ monomers undergo a slow “seeding”, nucleating phase into damaging Αβ oligomers, which then further aggregate into insoluble Aβ fibrils and diffuse amyloid plaques without hp-tau aggregates^3^. Misfolded hp-tau and Aβ can also destabilize their respective native conformations and spread equivalently as prions in conditions including increased acidity^3^. Moreover, Aβ and hp-tau pathology can promote seeding and further accumulation of the other (Fig. 1)^3^. While Aβ protein levels are generally increased in patients with LOAD *vs* controls, diffuse amyloid plaques and insoluble Aβ fibril aggregation do not correlate well with LOAD progression^1,120,125^.

The *Tau Hypothesis* suggests that upstream enzymes including p38 MAPK and GSK3 phosphorylate multiple residues in tau protein to create hyperphosphorylated tau (hp-tau)^1,3^. In this hypothesis, hp-tau starts as soluble monomers and oligomers but can form higher-complexity aggregates in insoluble fibrils, neuropil threads, and neurofibrillary tangles (NFTs). Neuritic plaques, which comprise compact amyloid plaques containing Aβ, hp-tau, and dystrophic neurites^1,3,106,125^, also constitute as hp-tau pathology. Together, all forms of hp-tau aggregation are classified as “neurofibrillary pathology”. Hp-tau or neurofibrillary pathology is considered to correlate well with cognitive impairment and dementia^1,100,125^.

**Fig. 2:**
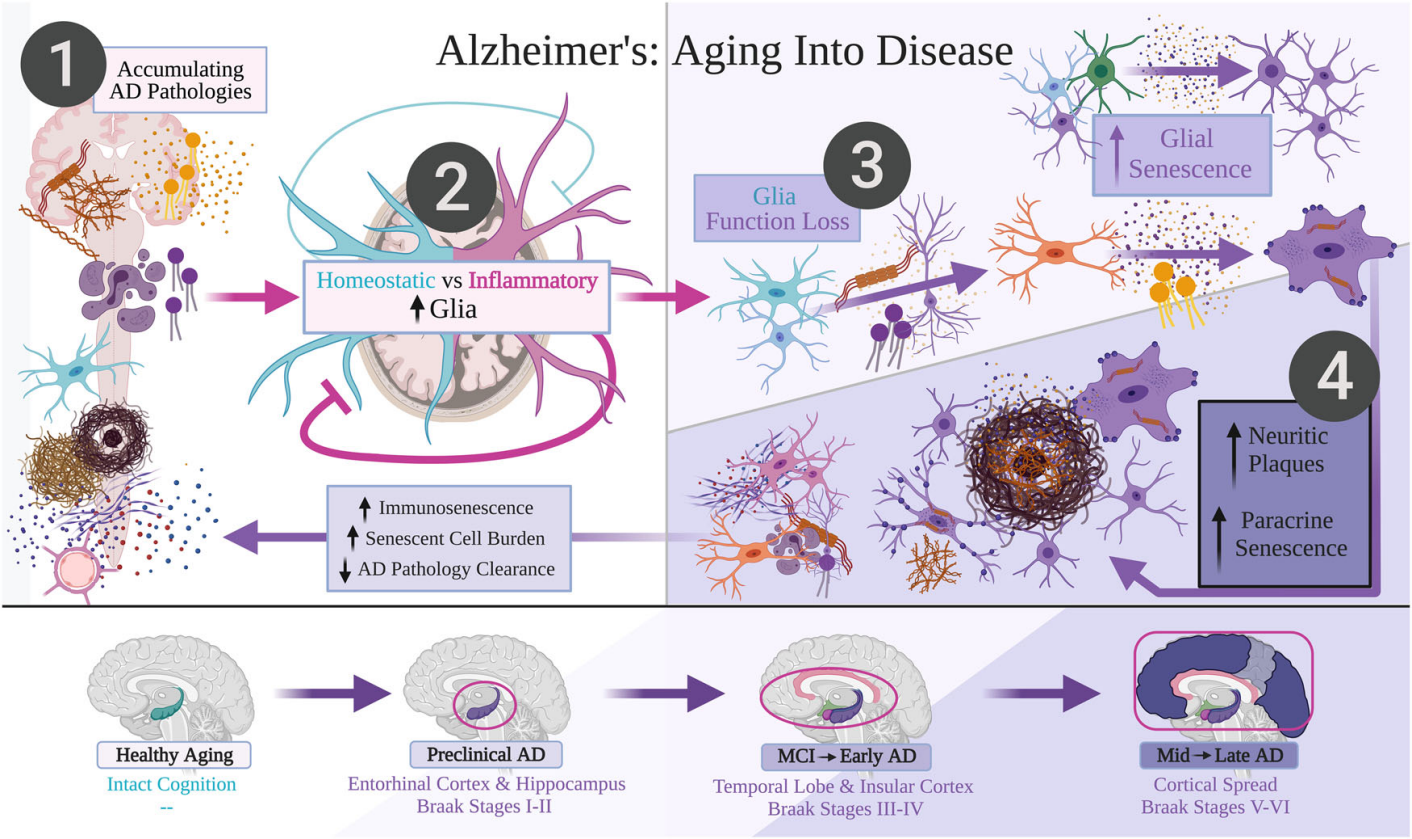
Alzheimer’s: aging into disease. A late-onset Alzheimer’s disease (LOAD) framework is proposed from healthy cognitive aging to late-stage Alzheimer’s disease (AD), presenting the following testable hypothesis: Glial senescence accumulation, particularly requiring increased senescent microglia burden, may correspond to clinical LOAD progression. Darker and deeper purple coloring denotes more severe senescent glia burden and Braak staging progression in LOAD. The hypothesis presents that: (i) Aging and AD pathologies from Fig. 1 (including amyloid-beta (Aβ), neurofibrillary pathology in hyper-phosphorylated tau (hp-tau), oxidative stress, and chronic inflammation) are predicted to progressively accrue throughout aging as by-products of central nervous system function and metabolism. (ii) Main AD pathology levels are likely enhanced by inflammatory glial states, and would be sufficiently cleared by glia performing homeostatic roles. However, homeostatic glial functions that putatively decline throughout aging would decrease proficiency in containing AD pathologies. (iii) In preclinical AD, increasing proportions of aged glia likely interact with AD pathologies to become senescent. This would include oligodendrocyte progenitor cells and astrocytes. Microglia performing homeostatic roles would also become senescent especially after engulfing neurons containing hp-tau. (iv) Senescent microglia then likely become incompetent in breaking down further phagocytosed hp-tau and Aβ aggregates, and instead secrete hp-tau to induce paracrine senescence in other microglia, especially those which are actively engulfing Aβ. Failure to suddenly degrade both hp-tau and Aβ likely induces secretion of Aβ and hp-tau aggregates that coalesce into neuritic amyloid plaques. These neuritic plaques correspond to Braak staging and clinical LOAD progression. Finally, this combination of senescent glia, reduced homeostatic glial support, and non-senescent, inflammatory glial states is predicted to drive neurodegeneration and cognitive decline in LOAD. While senescent glia burden is cleared out by immune cells, increasing immunosenescence over aging due to genetic and environmental circumstances likely slowly declines; thus, senescent glia burden is predicted to ultimately differentiate healthy cognition from mild cognitive impairment and LOAD. Figure created with BioRender.com.

*Braak Staging*: Post-mortem assessments of cognitive decline and correlates of neurodegeneration severity in LOAD have been extensively tracked via Braak staging^1,120^. Here, hp-tau aggregation and neurofibrillary degeneration are clinically documented into a set of six Braak stages^125^. In stages I and II, initial neurofibrillary pathology spreads from the transentorhinal region into the entorhinal cortex and hippocampus. Stages III and IV involve increased lesions and pathologies in these areas, as well as novel spread into the temporal lobe and insular cortex. Stages V and VI affect the remaining superior temporal gyrus and neocortex. Braak staging tracking hp-tau pathology burden was notably shown to correlate with LOAD progression, where stage IV is associated with an overt dementia diagnosis (Fig. 2)^1,100,125^.

*Hypotheses outside the Tau Hypothesis and Amyloid Cascade* *Hypothesis* focus on other contributors and risk factors associated with LOAD. Although this perspective hypothesizes that these factors eventually converge into increased glial senescence, comprehensive review of these theories is outside this perspective’s scope. Briefly, other factors include impaired metabolism via increased insulin, hypertension, obesity, and/or insufficient sleep^3,4^. Vascular dementia, consisting of progressive blood supply blockade, cerebral amyloid angiopathy (CAA) and Aβ deposition into the cerebral blood vessels, likely leads to and/or correlates with LOAD incidence^1,4^. AD pathology is often co-morbid with pathologies from other diseases such as Parkinson’s disease with dementia^1^. Finally, these different pathologies involve a neuroimmune component; inflammation caused by peripheral and central pro-inflammatory cytokines increases during LOAD, notably as a result of accumulating protein aggregation and compromised cell metabolism^2–4^.

## LOAD and aging

### Aging and oxidative stress

LOAD requires aging, during which aged cells accumulate reactive oxygen and nitrogen species (RONS);^21^ RONS accumulation can overwhelm antioxidant defenses to irreversibly alter and damage nucleotides, lipids, and proteins. This “oxidative stress” occurs repeatedly with age due to accumulating double-stranded DNA breaks, mitochondrial dysfunction, and declining efficiency of metabolic processes^5–7,21^. Oxidative stress activates pathways that elevate and/or accompany Aβ production, tau hyperphosphorylation, and release of pro-inflammatory cytokines including IL-1 and TNF^2,7,11,21,22^. With aging, increased pro-inflammatory cytokine production results in a chronic systemic “inflammation” that escalates over time, exacerbating RONS accumulation, cell damage, and leading to premature death^3,23^. LOAD risk factors including vascular pathology, sleep impairment, and chronic stress result in increased RONS levels as well as chronic, systemic inflammation^2–4^. Furthermore, increased inflammation and the NLRP3 inflammasome can induce local or paracrine senescence via IL-1α signaling^10^.

Oxidative stress damages all cell types including neurons. Particularly, RONS inhibit phosphatidylserine (PS) lipid translocases, increase intracellular calcium, deplete ATP, and activate phosphatidylserine scramblases; this “flips” PS from the inner to outer cell membrane, where exposed PS acts as an apoptotic or “eat-me” signal^24–26^. RONS create “oxysterols” by oxidizing membrane cholesterols; oxysterols are hypothesized to drive LOAD^27,28^. Oxidative stress acts via DNA damage and p38 MAPK signaling to induce senescence^7^.

In brain regions relevant to Braak staging, aging and AD pathologies can induce the accumulation of other pathologies causing further cellular damage and death in a circular relationship (Fig. 1)^2,3,5,6,11,21–23,28,29^.

### Aging and CNS glia

The CNS contains multiple glial types, including oligodendrocyte progenitor cells (OPCs), astrocytes, and microglia. Glia altogether serve numerous key roles^30^, and can be divided into “states” notably labeled as “homeostatic”. Homeostatic glial activities sequester and remove cellular products to preserve optimal function^30^, including degrading products associated with AD pathologies (Fig. 1)^31,32^. This parenchymal maintenance is achieved by paracrine signaling and endocytosis. Homeostatic glial functions involve release of trophic factors and anti-inflammatory cytokines (e.g., IL-10 and TGF-β), which acutely minimize cellular stress and pro-inflammatory reactions^33–35^. These molecules mediate communication in bi-directional loops of glia-neuron and glia-glia interactions, through which glia supporting one another facilitate neuronal activity and plasticity^30,34,36–40^. For instance, OPC-mediated functions via TGF-β2-TGFBR2-CX3CR1 signaling sustain homeostatic microglial activities^33,34^. Astrocytes also produce trophic factors and cytokines including IL-3 to reduce pro-inflammatory microglial states^41^, and both glia endocytose neuronal, synaptic, and extracellular elements^42–48^. Such endocytosed elements include the AD pathologies illustrated in Fig. 1;^3,30^ microglia phagocytose extracellular Aβ and tau through receptors like TREM2 and IGF1R^3^.

Receptors that facilitate Aβ and tau clearance can push homeostatic glia into “pro-inflammatory” states. Examples comprise RAGE and LRP1 receptors activating the NLRP3 inflammasome^3,49–51^. While pro-inflammatory glia can effectively protect against acute illness and infection, their continuous and exaggerated reactions during aging can instead promote cellular stress, premature death, and (indirectly) AD pathologies and neurodegeneration in patients with LOAD *vs* resilient individuals^2,3,5,52^. Gradually reducing homeostatic support for glia may equate to “priming”, where aged glia “hyperactivate” pro-inflammatory responses towards smaller amounts of pro-inflammatory cytokines or Aβ^11,53,54^. In parallel, aging and exposure to RONS reduce homeostatic signaling, notably impairing the TGFβII-Smad pathway in aged microglia^46,47,51^. Aged microglia and astrocytes can additionally decline in metabolism and endolysosomal systems^6,7,51,53,54^, likely resulting in reduced phagocytotic capacity leading to exacerbated Aβ and hp-tau deposition^54,55^. Thus, while homeostatic glial functions normally protect the adult brain, their ability to combat both increasing pro-inflammatory glial responses and traditional AD pathologies likely wanes with aging and risk factor exposure (Fig. 2).

### Aging and AD as putative inducers of glial senescence

**Fig. 3:**
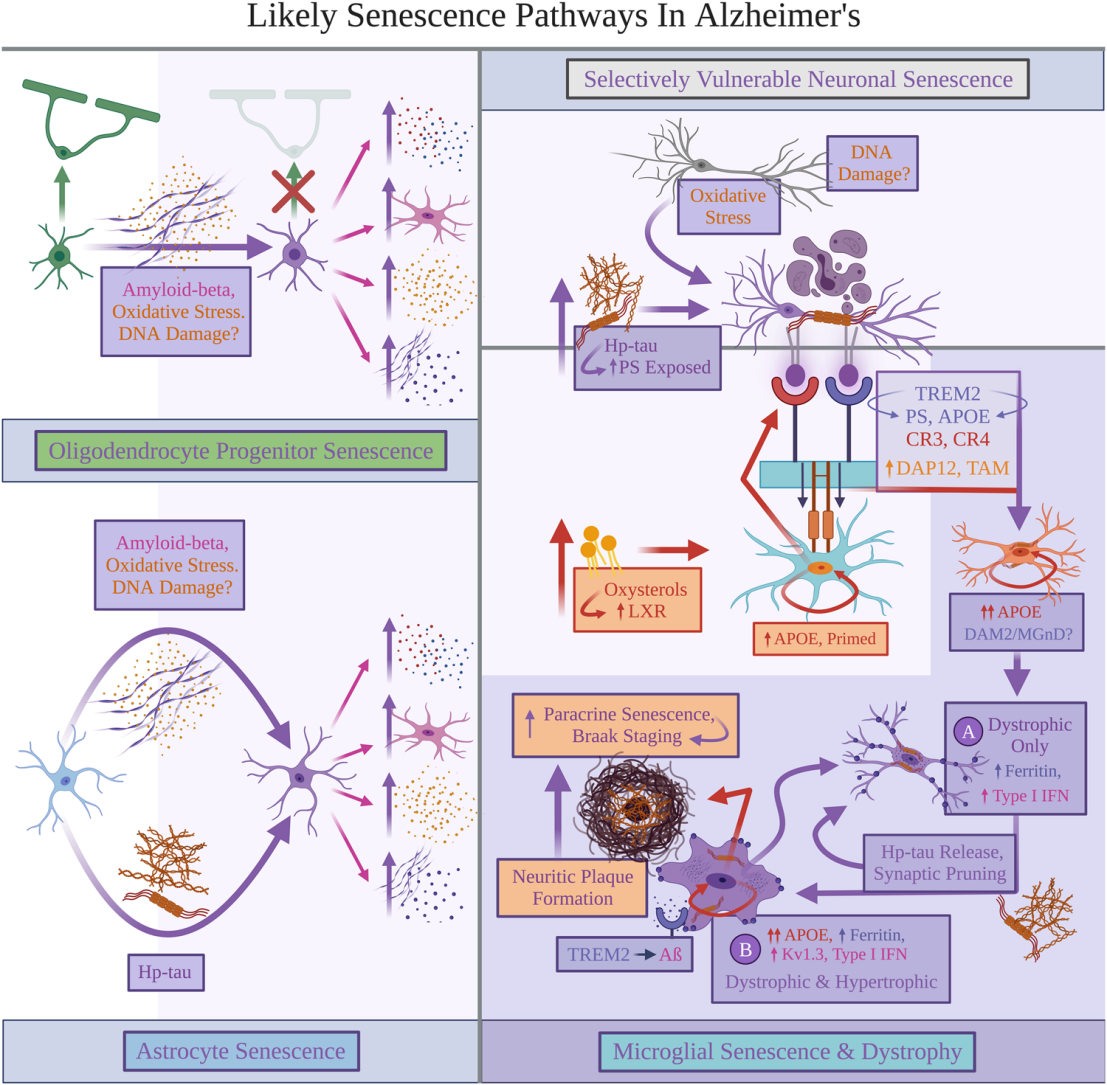
Likely senescence pathways in Alzheimer’s. Explanations for Alzheimer’s disease (AD) pathologies inducing senescence are proposed. Oligodendrocyte progenitor cells (OPCs) and astrocytes likely both react with amyloid-beta (Aβ) to become senescent. Oxidative stress and DNA damage may specifically lead to Aβ−induced senescence. Senescent OPCs and astrocytes are predicted to indirectly increase four pathologies: pro-inflammatory cytokines and reactive microglial states, oxidative stress, and Aβ accumulation. In neurons, oxidative stress, inflammation, and possibly DNA damage likely induce tau hyperphosphorylation; this process may also render the affected neurons senescent. Oxidative stress and hyperphosphorylated tau (hp-tau) aggregation likely prompt neurons to translocate and expose outer phosphatidylserine (PS). While aging predisposes sustained increases in local levels of inflammation, oxidative stress, and Aβ pathology burden, senescent OPCs and astrocytes are proposed to further worsen AD pathology. Senescent OPCs and astrocytes speculatively favor AD pathology buildup and environmental conditions that induce exacerbated microglial senescence in LOAD. Particularly, Aβ pathology contributes buildup to oxidative stress that oxidizes cholesterol to form oxysterols; these oxysterols may bind with liver-X-receptor expressed by homeostatic microglia states, resulting in upregulated apolipoprotein E (APOE) expression. Oxidative stress can also prime APOE-upregulated microglia to bind with neuronal PS using various receptors, enabling microglia to prematurely phagocytose and kill PS-exposed neurons containing hp-tau. This likely renders microglia senescent and unable to effectively phagocytose further AD pathologies, becoming proposed subtype or state “A” senescent microglia increasing type I interferon signaling, displaying dystrophic morphology, accumulating ferritin, and facilitating increased synaptic loss. They also proposedly secrete soluble hp-tau rendering paracrine senescence in nearby glia, thereby preceding neurofibrillary tangle accumulation in neurons. Local microglia attempting to actively phagocytose Aβ are predicted to be caught in this paracrine senescence feedback loop, becoming subtype or state “B” senescent microglia characterized by simultaneous dystrophy and a hypertrophic or “ameboid” appearance. We hypothesize that these hypertrophic, dystrophic, and senescent microglia secrete partially-digested aggregates that form neuritic plaques and further induce paracrine glial senescence. Finally, fewer homeostatic microglia proposedly remain available to degrade Aβ; Aβ would likely continuously accumulate and initiate increased pro-inflammatory responses, *APOE*, and *KCNA3* upregulation in state “B” senescent microglia. Figure created with BioRender.com.

As aging promotes oxidative stress and AD pathology, cells experiencing these stressors can be predisposed towards becoming senescent throughout one’s lifespan (Fig. 3.). In healthy physiology, immune cells including microglia phagocytose cells exposing PS^42^, which may include stressed and senescent cells^24^. However, immune cells can also decline over one’s lifespan and become senescent themselves. This “immunosenescence’” is offset by beneficial (epi)genetic variation and the ability of immune cells to renew by proliferation^18^. As there is a limit to mitotic cycles and environmental stresses that can be tolerated, immune cells can only proliferate a maximum number before becoming senescent. With increasing senescence and immunosenescence burden over time, senescence is considered a pathology of aging^7^.

Because LOAD is an aging disease involving senescence^1,3^, we propose that progressive cognitive decline and memory loss observed in LOAD are related to senescence. Indeed, shortened telomere lengths and increased immunosenescence are found in patients with LOAD^18,19^. Multiple mouse models displaying cognitive impairment and tissue dysfunction demonstrated senescent cells in the brain, where removing p16^INK4A^-upregulated, likely-senescent cells was able to reduce inflammation and increased performance in spatial memory tasks^15,17,39,56^. The aged vs younger mouse brain has higher levels of senescent cell markers^15,17^. This supports the view that LOAD-associated cognitive impairment progresses in an environment associated with increased senescent cell burden. Together, age-associated increases in oxidative stress, Aβ accumulation and hp-tau pathology likely induce senescence among glia.

In LOAD, APP overexpression and subsequent Aβ accumulation likely exacerbate OPC loss of function and senescence (Fig. 3). In APP/PS1 mice overexpressing Aβ, OPCs surrounding Aβ plaques positively stained for senescent markers p21, p16^INK4A^, and SA-β-gal^39^. Administering the antihistamine clemastine preserved OPC and oligodendrocyte function leading to restored myelin levels in 7–8 month APP/PS1 mice equivalent to those of age-matched, wild-type controls; clemastine also reduced SA-β-gal and p21-upregulated OPC numbers^57^. Lower OPC senescence may be tied to increased myelination in APP/PS1 mice; patients with LOAD exhibit decreased white matter integrity *vs* healthy controls^58^. 14-month APP/PS1 mouse hippocampi also display decreased OPCs and correlated myelin counts *vs* controls^59^, reinforcing that OPC senescence may contribute to the reduced oligodendrocyte differentiation and myelin integrity observed in patients with LOAD^57,58^. As senescent neural progenitors additionally release HMGB1 and inhibit OPC function leading to decreased remyelination^60^, glial senescence may simultaneously induce dysfunction in non-senescent, homeostatic OPC states.

NAD + levels deplete over aging and neurodegenerative disease progression, increasing cGAS-cGAMP-STING signaling and inflammation^61,62^. Hou et al. supplemented 7-month-old APP/PS1 mice with NAD + precursor (NR) for 5 months. In the cortex, NR inhibited cGAS-cGAMP-STING signaling, which alleviated both inflammation and senescence counts (via decreased p16^INK4A^-upregulated immunostaining)^62^. Removing senescent OPCs via senolytics, and decreasing senescent cell (including OPC) burden via NAD + supplementation, independently increased spatial memory in APP/PS1 mice^39,62^, with the treated APP/PS1 mice performing equivalently to wild-type controls. Targeting glial senescence and aging may likely improve cognitive impairment in mild cognitive impairment (MCI) and LOAD.

Zhang et al. investigated patients with LOAD vs diagnosed with MCI *vs* control subjects without dementia. Control subjects without dementia were categorized as Braak stages 0–I, patients with MCI as Braak stages III–V, and those with LOAD as Braak stage VI (exempting one Braak stage III–IV)^39^. Braak stage progression speculatively accompanies increased Aβ plaques, where patients with LOAD vs MCI had an average senescent OPC count per plaque significantly higher in the inferior parietal cortex; patients with MCI displayed a higher average, non-significant senescent OPC count *vs* control subjects without dementia^39^. We speculate that (i) OPC senescence begins with an increased Aβ burden in older age and precedes clinical dementia diagnosis, then (ii) continues to accumulate along the disease progression into latter Braak stages (Fig. 2). As Aβ contributes to increased RONS levels in neurons^23^, OPCs may potentially become senescent via DNA damage and/or oxidative stress (Fig. 3).

Astrocytes likely undergo increased senescence with AD pathologies. Oxidative stress, Aβ, and hp-tau can independently induce human astrocyte senescence in-vitro^63–65^. These senescent astrocytes exhibit a SASP profile, releasing pro-inflammatory cytokines and MMPs^63–65^. Senescent astrocytes induced by hp-tau uptake display a SASP profile and contribute to cognitive impairment via HMGB1 and NLRP3 inflammasome activation^29,64^. Their effects may contribute to driving AD pathology buildup, and negatively impact healthy functioning in other cells. Senescent astrocytes down-regulate Kir4.1 and EAAT1/2 transporters, leading to extracellular glutamate, neuronal excitotoxicity and cell death^36,38^, and increased NMDA-receptor signaling and pro-inflammatory microglial states^66^. Primary mouse astrocytes aged in-vitro display a SASP profile, where medium containing released extracellular vesicles from aged astrocytes (*vs* young astrocytes without a SASP profile) failed to support OPC differentiation^37^. As lacking OPC differentiation induces lower oligodendrocyte counts and myelin generation^57^, senescent astrocyte activity may promote decreased white matter integrity observed in patients with LOAD^58^.

Senescent astrocytes (p16^INK4^+) are abundant during human aging and neurodegenerative disease^38^, and display increased p16^INK4A^ and MMP-1 expression among the frontal cortex in LOAD^63^. Over 75% of GFAP + astrocytes were p16^INK4A^ + in the frontal cortex of patients with LOAD, and frequently stained for hp-tau oligomers and γ-H2AX indicating DNA damage^64^. Furthermore, p16^INK4^+ mouse astrocytic cells exposed to hp-tau in culture released extracellular HMGB1; this HMGB1 was collected as culture medium and induced further senescence in healthy astrocyte cultures via positive SA-β-gal staining, cell cycle arrest, and increased IL-6 and TNF release^64^. As senescent astrocytes likely induce dysfunction in other glia^36,38,66^, this dysfunction may involve paracrine senescence that transforms previously-homeostatic astrocytes^64^. HMGB1 exposure inhibited OPC differentiation and decreased myelination in rat spinal cord and human cultures derived from induced pluripotent stem cells;^60,67^ senescent astrocytes may release HMGB1 to induce OPC dysfunction and reduce white matter integrity in LOAD^58^.

Increased senescent OPCs and astrocytes may together exacerbate AD pathology burden and critically contribute to LOAD (Fig. 3). We hypothesize that senescent glia promote AD pathologies (Fig. 1) by weakening neuronal support and triggering cell death, notably via RONS release and NLRP3 inflammasome activation^3,23,29,50^, and further inducing pro-inflammatory glial states^5,6^. Astrocyte and OPC senescence may also induce paracrine glial senescence through RONS release and NLRP3 activation via IL-1α signaling^5,10,19^. However, while Aβ and/or likely oxidative stress can trigger OPC and astrocyte senescence without hp-tau involvement^39,59,65^, astrocyte and OPC senescence are not confirmed yet to correlate with neurofibrillary degeneration in Braak staging. Thus, an “additional player” could be needed to predict how glial senescence may mediate a preclinical to clinical LOAD transition.

We speculate that in preclinical LOAD, senescent OPCs and astrocytes withdraw their homeostatic support and exacerbate AD pathology accumulation. These effects are posited to “prime” homeostatic microglial states into becoming mainly pro-inflammatory and phagocytosing more neurons^5,6,33,34,41,51,53,66^, and may provide local environments that strongly favor microglial senescence–considered here as the “additional factor”. Senescent microglia abundance is argued as the final requirement for a preclinical to clinical LOAD transition, by potentially accelerating neurofibrillary pathology spread (Figs. 2, 3).

### Local microglial senescence accumulation in LOAD

#### Tau and type I IFN responses in microglial senescence

When microglia are primed to phagocytose in LOAD, they likely uptake hp-tau aggregates and then become senescent. In a P301S tauopathy mouse model overproducing hyperphosphorylated human tau, microglia displayed p16^INK4A^ and SA-β-gal staining^56^. Microglial states associated with neurodegeneration were similarly observed in P301S and P301L mice (also overproducing human hp-tau)^68^, where these microglia initially upregulated pro-inflammatory genes. These microglia later transitioned into populations displaying gene enrichment linked to senescence, such as inhibited cell cycle, upregulated type I IFN release^5–7,14^, and reduced pro-inflammatory signaling^68^. Thus, we speculate increased local concentrations of hp-tau aggregates could induce microglial senescence.

In-vitro tau uptake by primary mouse microglia caused cytoplasmic binding to intracellular PQBP1, PQBP1-cGAS-cGAMP-STING signaling, and an initial inflammatory response^69^. This work also provided in-vivo confirmation of PQBP1-tau colocalization in R6/2 transgenic mice modeling Huntington’s disease pathology. Using an immunodeficient mouse model with human iPSC-derived microglia from patients with Down syndrome, Jin et al. added tissue containing hp-tau from patients diagnosed with both LOAD and Down syndrome; exposed human microglia were highly enriched in genes implicating senescence, type I IFN release and signaling^70^. They also found that type I IFN-α/β receptor (IFNAR) knockdown restored impaired synaptic plasticity and neurotransmission, concluding that increased type I IFN release and signaling in microglia exacerbated synaptic pruning^70^. Senescent microglia were found to display (i) upregulated type I IFN release, by which they (ii) likely mediate increased synapse loss—a central mechanism in LOAD progression and pathology^1,2^. This explanation aligns with the findings of synapse loss and microglial reactivity preceding NFT formation in P301S mice^71^.

APP/PS1 and 5xFAD mice feature upregulated microglial type I IFN signaling and increased synaptic loss;^72,73^ ablating microglia-specific IFNAR also reduced post-synaptic loss in 5xFAD mice^73^. 5xFAD mice notably demonstrate hp-tau staining^73^, particularly in neuritic plaques^74^. By blocking IFNAR and subsequent type I IFN signaling in these mice, interferon and inflammation mRNA levels in 5xFAD hippocampi were lowered; IFNAR inhibition further restored spatial memory and synaptic deficits in 5xFAD mice to levels equivalent to wild-type controls^73^. This complements the findings by Jin et al.^70^, where senescent microglia were shown to upregulate type I IFN signaling and increase synaptic loss via pruning. Furthermore, IFNAR blockade did not alleviate Aβ burden in 5xFAD mice^73^. This supports our prediction that senescent microglia accumulation within key brain regions may determine cognitive impairment and LOAD progression (Figs. 1, 2).

Microglia may selectively become senescent after engulfing hp-tau through their capacity to prematurely kill neurons via phagocytosis, or “phagoptosis” (Box 2). Both extracellular and intracellular hp-tau inducing PS exposure in neurons were shown to lead to microglia phagoptosing neurons in-vitro^75,76^. In mouse microglial and P301S neuronal co-cultures, microglia phagoptosed neurons containing hp-tau aggregates^77^. These microglia became senescent via increased NF-κβ activation, MMP-3 release, and positive SA-β-gal staining; these senescent microglia also reduced their phagocytic capacity and phagoptosis of additional P301S neurons containing hp-tau, while further secreting insoluble hp-tau aggregates^77^. As cultured human microglial cells can also phagoptose PS-exposed, P301S neurons containing hp-tau aggregates^57^, both human and mouse senescent microglia can likely release and seed insoluble hp-tau aggregates^78–80^. As senescent microglia cannot remove these neurons, however, increased microglial senescence could indirectly permit increased NFT formation.

Conditioned media isolated from senescent microglia and P301S neuronal co-cultures induced microglial senescence in separate wild-type mouse cultures;^77^ thus, senescent microglial secretions containing hp-tau triggered local and paracrine, microglial senescence^77^. Phagotopsis of neurons containing hp-tau aggregates could therefore precede hp-tau-mediated, paracrine senescence^77^, although these processes are likely inevitably coupled over LOAD progression. Hp-tau seeding and release by senescent microglia putatively induce a pathological cycle of selective neuronal vulnerability to tau hyperphosphorylation, neuronal phagoptosis, synaptic loss, and paracrine senescence^70,72,75–77^ (Fig. 3).

#### Microglial priming and senescence in LOAD

As hypothesized above, LOAD would likely require glial aging and AD pathology buildup to induce microglial priming and senescence. We will further speculate here that microglial priming in LOAD involves increased oxidative stress and accelerated oxysterol production over aging^27,28^. Oxysterol accumulation is recognized by microglial liver X receptor (LXR), whereupon oxysterol and LXR binding, microglial *APOE* is upregulated^81^. In AD, upregulated APOE and “primed” microglia may correspond to DAM1 in mouse models^82,83^ (Box 2). While potential functions remain unclear, this may induce APOE secretion; as oxysterols deplete unoxidized cholesterol, LXR-stimulated microglia may secrete APOE packaged with cholesterol to aid in neuronal, myelin, and synaptic phagocytosis^84^. Upregulated *APOE3/4* can act as a nuclear receptor in microglia (Fig. 3) to accelerate their aging and pro-inflammatory responses, as well as increase Aβ aggregation, tau hyperphosphorylation, and phagoptosis in *APOE4* variants^85–87^. APOE secretion also leads to APOE-PS binding to stressed, desialylated neurons^25^.

We hypothesize that local environments speculatively promoting microglial APOE may (i) further pressure neurons to expose outer PS and accumulate tau hyperphosphorylation, and (ii) prime particular microglial states to phagotopse neurons (Figs. 2, 3). Reinforcing earlier discussion on hp-tau aggregates and neuronal phagoptosis, *APOE* knockdown in P301S mice significantly reduced hippocampal and entorhinal cortex loss;^40^ APOE preservation increased Iba1 + (suggesting microglia and macrophage populations) cells that were positive with CD68 + phagocytic inclusions^40,88^. As Shi et al. also found that astrocytes secreted APOE in P301S mice, they investigated whether APOE contributions to neurodegeneration and advanced hp-tau deposition resulted from astrocytes or microglia. Depleting a majority of microglia via CSF1R inhibition in P301S mice equally preserved the hippocampus and entorhinal cortex, while leaving astrocytic numbers significantly unaffected^40^. As microglial ablation blocked advanced deposition of hp-tau pathology, microglia and APOE were concluded to synergistically exacerbate hp-tau pathology progression and neurodegeneration^40^. APOE likely critically primes microglia towards advancing LOAD progression, suggesting that senescent microglia, involving hp-tau specificity, are likely a “final” requirement needed to advance neurofibrillary pathology and LOAD progression.

Homeostatic microglial states expressing TREM2 and upregulating *APOE* are predicted to bind PS-exposed neurons with hp-tau aggregates (Fig. 3), likely triggering neuronal and hp-tau phagoptosis via TREM2, complement receptors and opsonins^25^. It is currently unknown whether a TREM2-dependent, DAM2 program itself initiates senescence, or if phagoptosing neurons with hp-tau induces simultaneous DAM2 and senescence. A DAM2(-like) microglia state can likely overlap with senescence; DAM2 transcriptomic signatures are separately enriched in senescent microglia in aging, tauopathy and Aβ-overproducing transgenic mouse models, and in senescent human microglia exposed to LOAD patient tissue containing hp-tau^17,68,70,89^.

Mechanisms inducing senescence via hp-tau are currently unclear, but hp-tau may initiate senescence to inhibit apoptosis^90^. NFT-containing neurons from P301L mice and patients with frontotemporal dementia displayed upregulated senescence markers including *CDK2NA*, *TNF*, and *IL-1β* ^5,91^. Neurons may moreover induce senescence to prevent apoptosis triggered by oxidative stress and DNA damage^8,90,92^. Vulnerable neurons affected in Braak staging are often excitatory and display NFT formation^93,94^. As hp-tau induces microglial and astrocytic senescence^56,64,77^, “stressed” neurons exposed to ROS may become senescent through oxidative-stress-induced, tau hyperphosphorylation (Fig. 3). These neurons may then expose PS^75^, prior to rendering microglia senescent. Together, hp-tau pathology could induce senescence among multiple cell types (Fig. 3).

Furthermore, single-cell transcriptomic studies indicate that microglia display APOE priming and senescence in patients with LOAD. Olah et al. found that transcriptomic clusters 7, 8, and 4 were particularly enriched in patients with LOAD *vs* controls^95^. All three microglial clusters upregulated *APOE;* aged and *APOE*-primed microglial states may represent transcriptomic cluster 7, where microglial states in clusters 8 and 4 further displayed increased ferritin in *FTH1* and *FTL*^95^. As TREM2-involved phagoptosis is predicted to jointly induce microglial senescence and a DAM2(-like) microglial transcriptome^82^, cluster 8 microglia may speculatively represent newly-senescent microglia having uptaken hp-tau aggregates.

Cluster 4 microglia were enriched in multiple *IRF* transcription factors^95^ that suggest increased type I IFN signaling. Type I IFN signaling and synaptic loss are likely mediated via senescent microglia^72,96,97^, and are associated with plaque-associated microglia in samples from patients with LOAD^72^. An increased type I IFN release may sustain pro-inflammatory microglial reactions specifically to Aβ pathology. As senescent cells uniquely upregulate ferritin indicating iron accumulation^12,13^, senescent microglia may be enriched in LOAD. P301S and P301L mouse microglial transcriptomic signatures presenting cell cycle inhibition and type I IFN upregulation were also preserved in prefrontal and temporal cortex samples from patients with LOAD^68^. Genes enriched in type I IFN signaling were additionally upregulated and positively correlated with increasing Braak staging in parahippocampal gyri samples from patients with LOAD^72^.

A microglial population enriched in samples from patients with LOAD was also validated transcriptionally and via immunohistochemistry, exhibiting increased ferritin *FTH* and *FTL* expression, association with neurofibrillary pathology and particularly neuritic plaques, and a dystrophic morphology suggesting senescence^98^. This “dystrophic” microglial population was speculated to represent an “end result” microglial state responding to Aβ accumulation; these Aβ-responding microglia also upregulated *APOE*^98^. Finally, multiple microglial clusters were enriched in prefrontal cortex samples from patients with LOAD *vs* controls, demonstrating upregulated genes related to cellular senescence, including *IRF* transcription factors and *FTH1/FTL*^99^.

Box 2: Changes in microglial priming, ramified morphology, and phagoptosis relevant to LOAD While microglia in homeostatic states often display a ramified morphology, this morphological state does not always coincide with homeostatic functions in humans. Pro-inflammatory lipopolysaccharide induces a complex, hyper-ramified morphology in human microglial cells in-vitro^124^. Pro-inflammatory microglial states are also not necessarily equivalent to the “reactive” or hypertrophic, ameboid-shaped microglia containing phagocytic inclusions and clustering around neuritic plaques in samples from patients with LOAD;^88,110,123^ however, ameboid or hypertrophic morphologies can suggest that human microglia have phagocytosed extracellular materials^88,110^. This hypertrophic appearance is likely observed regardless of the microglia’s inflammatory status, as later discussed.

In normal physiological conditions, mouse microglia utilize TREM2 in phagocytosing synapses and whole neurons during CNS development, plasticity, and maintenance^26,42^. While homeostatic microglia can phagocytose compartments from dead neurons to optimize the surrounding environment, they can also phagocytose neurons that are stressed but not yet apoptotic. This process is referred to as “phagoptosis”, a premature, non-apoptotic death through microglial phagocytosis^24,25^. Mechanistically, oxidative stress exposes PS at the neuron’s outer cellular membrane to act as a ligand. Simultaneously, microglia reacting to pro-inflammatory stimuli produce sialidase that removes or “desialylates” neuronal sialic acids^24,25^. While sialic acids protect neurons from being phagocytosed^24,25^, microglia enter pro-inflammatory states more frequently during aging. Thus, aging likely renders neurons more susceptible to phagoptosis. Microglia in pro-inflammatory states may not directly partake in phagoptosis while secreting pro-inflammatory cytokines, but the resulting increases in oxidative stress may “prime” homeostatic microglia to phagoptose neurons. Exposure to extracellular hp-tau aggregates and generation of intracellular hp-tau complexes, respectively, could also prompt neurons to expose outer PS^75,76^.

Once neuronal PS is sufficiently exposed, extracellular opsonin proteins can coat outer PS and induce phagoptotic death by microglia^24,25^. This process occurs via multiple ligand–receptor pairs that converge into the DAP12/TYROBP/KARAP pathway, stimulating downstream signaling and subsequent phagocytosis through the TAM receptor tyrosine kinases Mer and Axl^24,25^. Specific pathways leading into phagoptosis and DAP12 signaling include TREM2-PS direct binding, or TREM2 binding to an extracellular APOE-PS complex^25,43–45,142^. Complement C1q is another opsonin that coats PS, allowing for subsequent C3b, iC3b complement binding and phagoptosis by CR1/CR3/CR4 complement receptors^25^. These complement receptors trigger downstream DAP12 signaling in microglia^24,25^. A separate pathway involves binding of the calreticulin opsonin to C1q, resulting in the phagoptosis of neurons by microglial LRP1, together with the induction of more pro-inflammatory, microglial responses^3,25^.

In mice, homeostatic microglial states can phagoptose neurons and correspondingly exhibit a neurodegeneration/disease-associated microglial state (DAM or MGnD)^82,83^. The transcriptome includes an initial DAM1 state, involving *APOE* upregulation independently of phagoptosis activation, and a subsequent DAM2 state; DAM2 necessitates TREM2-dependent phagoptosis, and further upregulates *APOE* and *ITGAX* relating to the CR4 subunit CD11c^82^. Further DAM2 substates were identified, with a pro-inflammatory substate upregulating the Kv1.3 channel protein mediating increased RONS and pro-inflammatory cytokine release^83^. Dark microglia represent another relevant state, observed using electron microscopy in mouse models of chronic stress, aging and Aβ pathology, as well as in aging individuals and patients with schizophrenia^119,143^. While more research is warranted to study this association, dark microglia display ultrastructural markers of oxidative stress and lipofuscin related to senescence. These cells could hypothetically participate in phagoptosis; dark microglia have increased phagocytic inclusions and commonly enwrap processes around shrunken, but still viable neuronal elements^119^.

### Predicted features of senescent microglia in LOAD

Patients with LOAD present dystrophic microglia^88,100–102^. While dystrophic microglia have been scored with different criteria, we will define an exclusive set of criterion for dystrophic microglia as a minimum requirement of (i) ferritin enrichment and (ii) progressive reduction in microglial process complexity; additional signs of (iii) spheroidal swellings and (iv) cytoplasmic deterioration in human dystrophic microglia likely only occur in advanced and/or late-stage dystrophy^70,102–104^. Dystrophic microglia associate with and precede NFT pathology following Braak staging^88,100–102^. These microglia not only increase in numbers over aging, but also in differing types of dementias vs age-matched healthy controls, notably in the hippocampus^104,105^.

In samples from patients with LOAD, dystrophic microglia lacking extremely hypertrophic somas (linked to increased reactivity) associate with neuritic plaques, neurofibrillary tangles, and dystrophic neurites positive for hp-tau even in the absence of Aβ aggregates^100,106^. Dystrophic microglia also contain hp-tau in aged tree shrews^107^, and are thus considered to correlate well with neurofibrillary burden^108^. Dystrophic microglia showing extreme hypertrophy or “ameboid” shapes associate with neuritic plaques in humans and marmosets;^88,109^ hypertrophic microglia clustering around neuritic plaques were observed in prefrontal cortex samples from patients with LOAD; where clustering hypertrophic and dystrophic microglia were speculated to reflect the preclinical to clinical LOAD transition^88^. This complements examinations of parahippocampal cortex samples from patients with LOAD, where microglia were concluded to transform from a “primed” or ramified appearance into hypertrophic or ameboid morphologies nearby neuritic plaques^110^. When microglia were investigated with a semi-quantitative “dystrophy” score, samples from the left anterior frontal and temporal lobes of patients with LOAD *vs* controls featured increased microglial dystrophy^104^.

Potential heterogeneity of microglial senescence remains to be rigorously confirmed with multiple markers in clinical LOAD. Nonetheless, dystrophic microglia in samples from patients with LOAD present increased APOE, ferritin expression, and iron accumulation, suggesting they are senescent^98,102,105,107,111,112^. Notably, senescent cells selectively uptake and accumulate iron;^12,13^ microglia do not normally accumulate iron^105^. Primary mouse and SV40 human microglial cells exposed to high iron display a dystrophic morphology, iron accumulation, and became ineffective at phagocytosing Aβ^103,113^. This indicates that iron accumulation could causally induce microglial dystrophy. Both neuritic plaques and NFTs contain hp-tau-positive inclusions^1,2^. Since dystrophic microglial abundance and proximity are correlated well with this neurofibrillary pathology^100^, dystrophic microglia are likely senescent due to significant hp-tau exposure.

In a neuron-specific P301S human tauopathy model, mouse microglia exposed to hp-tau did not display beaded processes observed in human dystrophic microglia; they instead presented lysosomal swellings with hp-tau inclusions and reduced morphological complexity indicating dystrophy^102,114^. In-vivo mouse microglia exposed to hp-tau from LOAD patient tissue upregulated ferritin and displayed reduced morphological complexity and process length^70^. Multiple senescent cell types contain altered lysosomes with larger sizes, higher counts, and decreased function^7^. In a neuronal VPS35-knockout mouse model, increased APOE levels positively correlated with Iba1 levels indicating increased microglial reactivity. When assessing this reactivity in-situ, microglial dystrophy and endosome swelling were observed (indicating failure to break down products and endolysosomal impairment)^115^. Furthermore, this mouse model demonstrated increased ex-vivo tau secretion from neurons^116^. This reinforces that hp-tau may induce APOE upregulation, resulting in microglia with dystrophic and senescence traits.

Neumann et al. investigated brain samples from aged individuals categorized under Braak I-V, and quantified Iba1+ cell proportions positive for senescence markers across multiple brain regions; Iba1 staining was used to visualize microglial morphology and categorize these cells as dystrophic^117^. While proportions of Iba1+ dystrophic microglia displaying each senescence marker were not mentioned, several markers associated with senescence (lipofuscin accumulation, γ-H2AX upregulation, and lamin B1 downregulation^7^) were not concluded as overrepresented in human Iba1+ dystrophic vs ramified microglia^117^. It should be noted that senescent cells likely have high heterogeneity in their marker expression dependent on local environment, cell type, state and sub-state(s)^7^. These authors observed both dystrophic Iba1+/ferritin+ microglia and Iba1-/ferritin+ microglia. However, lipofuscin accumulation, γ-H2AX upregulation, and lamin B1 downregulation were not examined in Iba1-/ferritin+ microglia^117^.

Tischer et al. previously demonstrated that CD68 + inclusions visible in fluorescence microscopy exist between “fragmented” processes from Iba1+ dystrophic microglia^118^. Correlative light and electron microscopy revealed that these processes were not truly fragmented; rather, Iba1+ dystrophic microglia possessed thin processes devoid of visible Iba1 staining^118^. Although Iba1 absence in these processes could be due to their thin nature, dystrophic microglia could also exist along a spectrum featuring varying levels of Iba1 downregulation. The dark microglia observed ultrastructurally display decreased Iba1 staining vs non-dark microglia^119^.

Markers specific to microglial senescence are not yet standardized across the field. As Neumann et al. suggest, Iba1-/ferritin+ microglial states could be senescent and enriched for increased lipofuscin, γ-H2AX positivity, and/or downregulated lamin B1^117^. As reactive or ameboid morphologies were not examined^117^, simultaneously dystrophic and senescent microglia per our hypothesis may have been uninvestigated. Such hypertrophic senescent microglia may express some of the investigated senescence markers, based on their putative hypertrophy from numerous phagocytic inclusions (Fig. 3)^105,107,112^.

Finally, Jin et al. demonstrated that human and mouse dystrophic microglia are ferritin+ and enriched in senescence genes including *B2M*, ZFP36L1, and genes associated with type I IFN responses^70^. In-vivo human iPSC-derived microglia grafted into immunodeficient mice and exposed to LOAD samples containing soluble hp-tau displayed upregulated ferritin, interferon transcriptomic markers, and correlated with increased synaptic loss^70^. This in-vivo hp-tau exposure triggered dystrophic morphologies in human microglia^70^. As IFNAR knockdown also inhibited development of dystrophic morphologies and upregulated ferritin in these human microglia, even in response to hp-tau, this implies that sustained type I IFN release promotes senescence and iron accumulation in dystrophic microglia^70^. Senescent microglia likely release type I IFN to facilitate initial inflammation and synaptic loss^69,70,72,73^, then seed and spread hp-tau aggregates that induce paracrine senescence;^77^ dystrophic microglia thus may act via multiple mechanisms central in LOAD pathogenesis to promote neurodegeneration.

Neurofibrillary pathology includes neuritic plaque formation and accumulation, which correlates well with Braak staging, cognitive decline, and overall LOAD progression^1,120^. Neuritic plaques appeared before other hp-tau aggregate types both in human-tau-injected APP knock-in mice and samples from patients with LOAD; in these mice, neuritic plaque tau induced quicker seeding of neuropil threads and NFTs^121^. Notably, NLRP3 inflammasome staining and enriched gene transcripts related to type I IFN signaling were localized proximal to neuritic plaques in samples from patients with LOAD^51,72^. In 5xFAD mice, depleting most microglia using CSF1R inhibition reduced neuritic plaque deposition^122^. As employing smaller CSF1R inhibitor dosages decreased microglial proliferation and senescence in APP/PS1 mice^89^, microglial senescence likely accounts for increased neuritic plaque deposition. APP/PS1 mouse hippocampi featured higher Iba1+/SA-β-gal+ cell (suggesting senescent microglia) proportions around Aβ plaques *vs* wild-type controls^89^. More Iba1+ cells also stained for p16^INK4^+/p21+ and surrounded Aβ plaques in temporal cortex samples from patients with LOAD *vs* control subjects without dementia^89^.

Because dystrophic microglia are likely senescent and specifically associated with hp-tau pathology, we propose that these cells critically advance LOAD progression and Braak staging. Particularly, dystrophic and hypertrophic microglia associating with neuritic plaques are predicted to correspond with “plaque-associated” microglia. In LOAD, “plaque-associated” microglia are classified based on neuritic plaque appearance; human microglia in samples from patients with LOAD do not significantly associate with non-neuritic, diffuse amyloid plaques^123^.

Here, we posit that primed microglia observed in patients with LOAD initially comprise a (hyper-)ramified morphology^124^. After endocytosing hp-tau aggregates locally or via phagoptosis, senescent microglia proposedly adopt a state “A” with dystrophic features characterized by ferritin upregulation, increased type I IFN signaling, and reduced morphological process complexity^70^ (Fig. 3). These dystrophic microglia may further display Iba1 loss^117^. As senescent microglia are endolysosomally compromised and ineffective at removing hp-tau neurons^77^, we propose that senescent microglia may uptake, but fail to effectively degrade Aβ and hp-tau aggregates^7,44,77^. Continued microglial senescence and impaired endolysosomal function are predicted to neglect neuronal support and permit, if not encourage NFT formation^98,100,101,125^. This aligns with observations that dystrophic microglia can precede NFT pathology accumulation in LOAD^100^, while microglial reactivity and synaptic loss occur prior to NFT appearance in P301S mice^71^.

Dystrophic microglia are predicted to secrete hp-tau aggregates^77,126^, via exosomes containing hp-tau that are taken up by nearby neurons^127,128^. As extracellular hp-tau induces PS exposure in neurons^76^, senescent microglia likely sustain premature neurodegeneration by seeding hp-tau aggregates and triggering local phagoptosis^77^. Secreted hp-tau oligomers from senescent microglia also likely enter local astrocytes to induce additional paracrine senescence and hp-tau spread^64^. As aggregated hp-tau increases NLRP3 inflammasome activation and pro-inflammatory cytokine release^51,79^, senescent microglia could additionally drive indirect hp-tau aggregation via pro-inflammatory reactions^50,68,70,79^.

State “A” dystrophic microglia likely exacerbate synaptic loss and neurodegeneration through increased type I IFN release and signaling^70–73^. We predict that they also induce paracrine senescence in nearby microglia attempting to phagocytose and digest Aβ aggregates^98^. Multiple mechanisms may facilitate Aβ endocytosis, including a strong TREM2-Aβ affinity binding;^129^ because APOE binds to Aβ oligomers^81^, actively phagocytosing microglia may endocytose via the TREM2-APOE-Aβ pathway. As “ameboid” or hypertrophic microglia can perform active phagocytosis in humans (Box 2), human hypertrophic microglia busily phagocytosing Aβ proposedly could become senescent via paracrine senescence and hp-tau secretion by state “A” dystrophic microglia;^77^ the posited outcome is a state “B”, which is dystrophic and hypertrophic and enriched in ferritin (Fig. 3)^105,112^. State “B” senescent microglia may also display Iba1 loss and enrichment in senescence markers not found in other dystrophic microglial states^105,107,112^. To provide increased rigor in assessing dystrophy in hypertrophic microglia, future quantitative assessments could investigate ferritin enrichment, potential bulbous swellings, and morphological deterioration within the “reactive” microglial states observed in patients with LOAD and aged marmosets^88,98,109,110,112,123^.

While both states of senescent microglia likely have type I IFN responses^14,70,72,96,97,99^, state “B” is predicted to respond to exacerbated Aβ aggregation by upregulating pro-inflammatory cytokine secretion and *Kv1.3* expression further exacerbating local inflammation^96,103,130,131^. This contrasts conditions devoid of Aβ pathology, where microglial senescence states induced by hp-tau exposure do not display extended, pro-inflammatory responses^70,77^. Senescent microglia may correspond to the transcriptomic cluster 4 per reference^95^, featuring increased *FTH1* and *FTL* expression implicating iron accumulation and senescence^12,13,98,105,112^, *APOE* upregulation, and *IRF* transcription factors expression corresponding to increased type I IFN release^14,72,96,97^. In mouse models of Aβ pathology, cluster 4 may further represent a pro-inflammatory, TREM2-dependent DAM2 state observed in response to Aβ pathology^83^. Notably, TREM2 in hypertrophic and/or dystrophic microglial states could be concomitantly downregulated due to inflammation^132^.

While non-fibrillar, Aβ secretion may form protective diffuse plaques, we propose that state “B” senescent microglia fail to degrade endocytosed Aβ and instead secrete Aβ-hp-tau aggregates^1,49,78–80,133^. This agrees with positive correlations of dystrophic microglia density and acidic pH environments across samples from multiple cortical areas in patients with LOAD *vs* non-demented individuals^106,134^. Acidic pH may reliably correlate with dystrophic senescent microglia, as (i) human microglia and P301S mouse microglia lysosomes feature hp-tau inclusions^70,114^, (ii) P301S neuron and mouse microglial co-cultures result in hp-tau seeds being secreted by senescent microglia^77^, and (iii) mouse microglia can pack phagocytosed Aβ fibrils within lysosomes and secrete acidified Aβ fibrils to build Aβ plaques^47^. Senescent cell lysosomes also retain an acidic pH^7^.

As hp-tau spread can induce microglia senescence^77^, Aβ-hp-tau aggregate secretion is also predicted as specific to senescent microglia^47–49,79,135^. These secreted Aβ-hp-tau aggregates proposedly coalesce into neuritic plaques with dystrophic neurites via microglial TAM receptors^47,110,123^. Secreted Aβ-hp-tau aggregates may be deposited into blood vessels and contribute to CAA, indirectly causing LOAD progression^136^. As plaque-associated or state “B” senescent microglia are predicted to seed hp-tau^80^, secreted hp-tau and Aβ aggregates may be continuously uptaken by nearby microglia attempting to contain AD pathology spread^3^. This compensatory response may induce further microglial senescence^77,98^, neuritic plaque formation, and higher senescent microglia counts around neuritic plaques within localized areas^89^. “Plaque-associated” microglial clustering seen in LOAD is proposed to emerge here due to a positive feedback loop of likely paracrine senescence, aggregate seeding, and neuritic plaque formation by several state “B” senescent microglia^88,98,110,123^. Exacerbated Aβ and hp-tau seeding may induce further astrocyte and OPC senescence proximal to neuritic plaques (Fig. 2).

As neuritic plaques precede and seed neuropil thread and NFT formation^121^, senescent microglia and neuritic plaques are predicted to drive both hp-tau spread and cognitive decline in LOAD. Senescent microglia are putatively responsible for sustained Aβ, hp-tau, and inflammatory increases beyond a tolerance limit likely dependent on each individual’s physiology. In an aging CNS milieu, the accumulation of senescent microglia is posited to determine overall LOAD progression^1,50,51,79,110,123,125,137^. Microglia can additionally proliferate in response to accumulated Aβ pathology^48^, which can further accelerate transitions towards senescence through telomere shortening^5,6,20,89^. This mechanism may independently generate senescent microglia that exacerbate Aβ pathology, local inflammation, and hp-tau secretion, indirectly increasing senescent microglia accumulation and hp-tau spread. However, while Aβ aggregation, DNA damage and other triggers may contribute to microglial senescence via replicative senescence or oxidative stress^20,89^, APP-overexpressing mouse models feature relatively decreased hp-tau pathology and senescent microglia abundance^56,77,89^. While encouraging experimental validation, this hypothesis reinforces that hp-tau aggregate uptake is likely specific and central towards triggering microglial senescence, neurofibrillary pathology, and ultimately LOAD progression^48,100,114,126,127,138^.

Microglial senescence likely occurs over one’s lifespan, but is not exclusive to LOAD; senescent microglia could be underrepresented or overrepresented among specific brain regions unrelated to Braak staging. We specifically propose here that buildup and decreased clearance of senescent microglia, within Braak staging-relevant regions, putatively underlie LOAD progression. Furthermore, although immune cells that include microglia can phagocytose and remove senescent cells^42^, immunosenescence also builds up over aging and likely predisposes a tissue environment for increased glial senescence burden^5,18^ (Fig. 2). As microglia are innate immune cells, microglial senescence may act as both (i) a final requirement for Braak staging progression and cognitive decline, and (ii) a final maladaptive form of immunosenescence. In LOAD, microglial clustering, presumed formation of neuritic plaques, and extreme hypertrophy among the prefrontal cortex emerge together with the clinical presentation around Braak stages III–IV^1,125^. Senescent microglia are thus predicted to actively accelerate hp-tau seeding and the build-up of neuritic plaques tipping preclinical LOAD into clinical progression^121,133^.

We hypothesize overall that buildup and clustering of senescent microglia (i) exacerbates accumulation of Aβ and neurofibrillary pathology, (ii) increases synaptic loss, pro-inflammatory glial responses, and paracrine senescence, and (iii) facilitates local neurodegenerative spread that results in clinical LOAD progression (Fig. 2)^52,137^. Microglial clustering around neuritic plaques can happen in local areas before any major neurodegeneration;^88,106^ samples from patients with LOAD demonstrated more microglial hypertrophy and clustering in relatively-spared primary motor cortex vs severely-affected inferior temporal cortex^106^. We thus predict that local increases in dystrophic microglia and their cumulative effects, including paracrine senescence, may vary between individuals and shape the heterogeneous progression of Braak staging in LOAD^1,137^.

Finally, we predict that the accumulation of senescent glia and dystrophic microglia differentiates the outcome between patients with LOAD vs resilient individuals with significant AD pathology, and healthy individuals without notable AD pathology^52^.

## Conclusion

In this perspective, we hypothesize that for healthy cognition to transition into MCI and LOAD diagnosis, (i–ii) aging eventually leads into AD pathology buildup that (iii) induces initial glial senescence, where (iv) subsequent paracrine senescence results in a positive feedback loop between all components (i–iv) (Figs. 1, 3). Localized paracrine senescence may vary individually and partially underlie the heterogeneous progression of LOAD^1,137^. Local, senescent microglia accumulation represents a central consideration in understanding and treating LOAD. Particularly, we predict that patients with LOAD progress into dementia due to senescent glia, dystrophic microglia, and neuritic plaque burden; we propose that dystrophic microglia burden is minimized in resilient individuals with significant AD pathology and healthy individuals without notable AD pathology^52^. As everyone has a unique cognitive reserve formed through individual physiology, genetics, and environmental experiences, a differing threshold number of senescent microglia may be needed to overwhelm each person’s cognitive reserve. An absolute number of dystrophic and senescent microglia within regions specific to Braak staging may insufficiently cause LOAD in one individual, yet possibly drive dementia progression in another.

Although this hypothesis is compatible with current LOAD explanations, our speculations and predictions should be rigorously assessed for validation or rejection. As we have not covered endothelial cell senescence and blood–brain barrier leakage, other LOAD risk factors, and potential contributions of senescent glia in mixed dementias and co-morbidities, this hypothesis can be expanded upon with future and independent reviews. We also focus on LOAD here, but mutations responsible for EOAD may involve quicker microglial senescence through exhaustion. Particularly, TREM2 R47H microglia may have decreased metabolic capacity to rapidly clear out Aβ and consequent hp-tau buildup in EOAD^20,139^. Conjecturally, mutations could make it harder for immune cells to clear out senescent microglial accumulation over aging^5^. Peripheral immune cells, including those in the innate and adaptive immune systems, may also become senescent and contribute to LOAD. There is currently insufficient evidence that infiltrating, non-macrophage populations become senescent and contribute towards AD pathology buildup and spread. It is, however, possible that dystrophic “microglia” (based on markers used to study them) can comprise both yolk-sac derived resident microglia and monocyte-derived macrophages infiltrating the CNS.

Senescent glia, including dystrophic microglia, likely comprise diverse morphologies and molecular signatures. As measuring these morphological and molecular states is not currently standardized across the field, we recommend confirming or rejecting the current framework by assessing microglial senescence in samples from patients with LOAD using multiple methods: ferritin enrichment, iron accumulation, acidic pH and/or SA-β-gal quantification, upregulated gene modules implicating senescence, and lipofuscin accumulation associated with state “B” microglia^5,7,89^. We also argue that a more universal criteria for dystrophic microglia should be incorporated, utilizing the criteria we proposed (when emphasizing measuring heterogeneity in dystrophy): (i) sustained ferritin enrichment, (ii) reduced process complexity, and optional indications of advanced dystrophy in (iii) spheroidal swellings and (iv) cytoplasmic deterioration especially in humans^70,102–104^. Although not methodologically feasible yet, strongly rejecting our hypothesis would involve tracking over time cognitive decline and potential increases in dystrophic microglia burden within the same individuals. We believe that this perspective currently offers explanatory power concerning possible dystrophic and senescent microglial contributions to AD pathology^70,72,73,77,88,99,100,105,111^, and hope that it will potentially shape future, therapeutic efforts for LOAD.

**Fig. 4:**
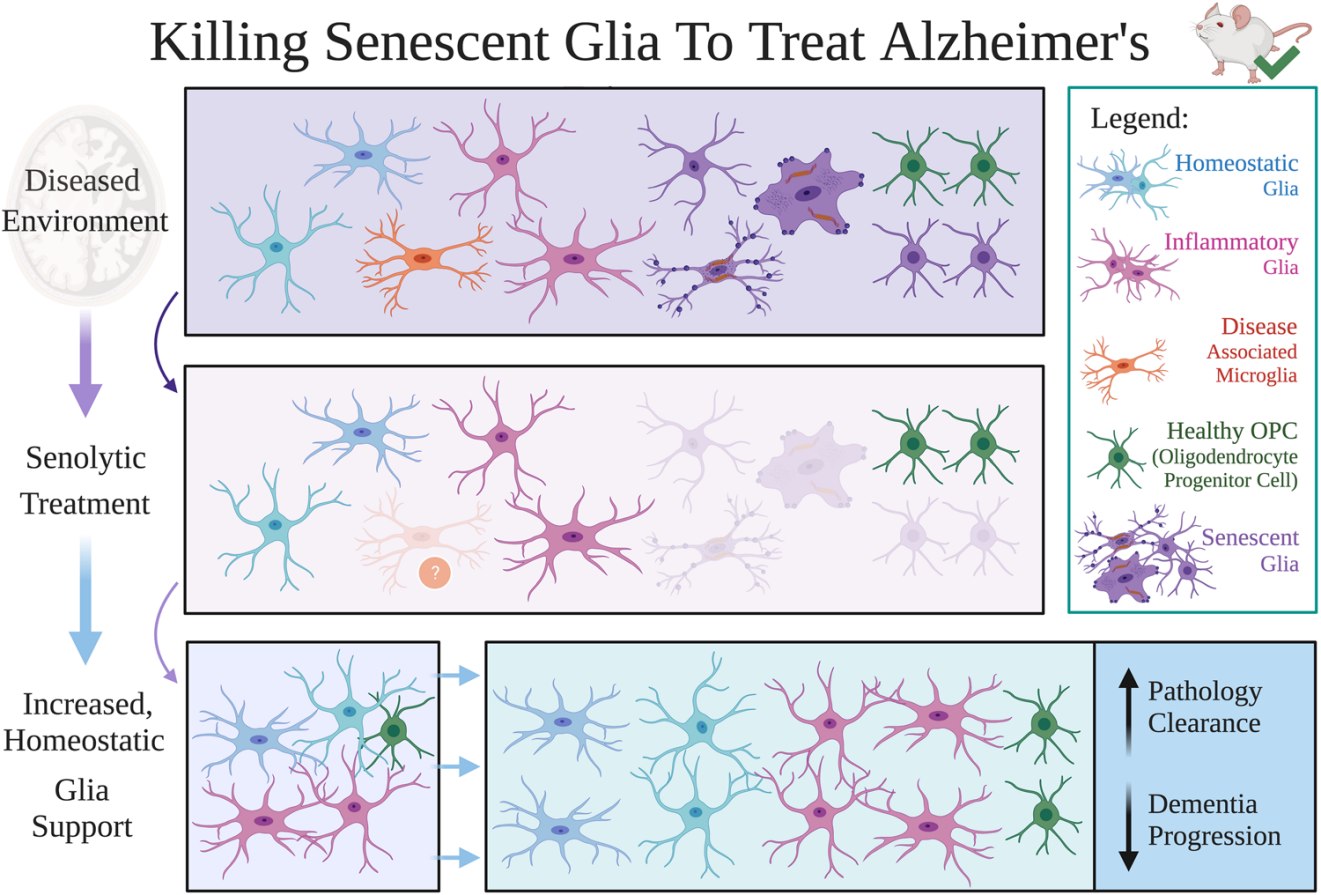
Killing senescent glia to treat Alzheimer’s disease. Building on evidence collected from studies involving mouse models, we conceptually provide a hypothesized outcome of using senolytics to treat Alzheimer’s disease (AD). Senolytics are drugs that selectively kill senescent cells, and were shown to reduce cognitive decline in disease mouse models. If senescent glia sustain synaptic loss and accumulation of the main AD pathologies, senolytics are predicted to provide a best chance for optimal outcomes in individuals with subjective cognitive decline, mild cognitive impairment, and early to mid-stage dementia. Alternatively put, senolytic interventions may best target earlier timepoints across the cognitive aging axis when one has a larger brain reserve, as late-stage AD may have already resulted in too much neurodegeneration and cell death. Remaining glial populations are predicted to repopulate and renew glial homeostatic functions to effectively clear out and reduce main AD pathologies, as reduced senescent glia counts should critically reduce AD pathology accumulation. Resulting plasticity should hopefully revert the aging brain back to a healthy cognition or preclinical late-onset AD (LOAD) state; minimally, we predict that optimized senolytic treatments can halt further LOAD progression at least transiently after administration. We also highly support the combination of other pharmacological and non-pharmacological treatment strategies with senolytics. This would hypothetically allow for better restoring of glial homeostatic functions, and would minimize damage accrued by pro-inflammatory glial states and other AD pathologies. The question mark denotes that it is currently unknown whether disease-associated microglial states are targeted by senolytics. Figure created with BioRender.com.

AD pathology is often co-morbid with other dementias and tauopathies^1^. Solutions targeting senescent glia in LOAD may likely target other disease outcomes; co-morbid pathologies may further involve independent mechanisms inducing glial cell senescence. Positron emission tomography imaging also supports that tau seeding occurs locally from Braak stages III+^137^. Thus, treating hp-tau seeding via potential senescent microglia targeting may also promote cognitive health along healthy aged individuals, by preventing MCI and age-related, neurodegenerative diseases. If this hypothesis is correct, increasing glial senescence could potentially account for negative previous clinical trial outcomes. As previous clinical trial candidates have not directly killed senescent glia, these treatments were likely unable to significantly halt the AD pathology accumulation sustained by senescent glia buildup. A specific solution towards treating LOAD is encouraged here through senolytics, or drugs that selectively kill senescent cells (Fig. 4)^140^. Successful senolytic administration is expected to treat the accelerated, traditional AD pathology burden caused by senescent glia, hopefully allowing renewal and recovery of remaining brain parenchyma to increase the quality of life in affected individuals. Using senolytics to treat MCI or early to mid-stage dementia prognosis would directly test our hypothesis, as senescent cell removal is predicted to halt the different proposed, pathological mechanisms propagated by senescent glia.

If senolytics alone are insufficient for stopping LOAD, we predict still that senolytics are necessary as co-treatment with other therapeutics. As senescent cells display apoptosis resistance^7^, probable senescent glia-mediated contributions to AD pathologies likely cannot be targeted only with non-senolytics. Albeit, sole senolytic administration has successfully treated and prevented cognitive impairment in tauopathy, amyloid, and aged mouse models^15,39,56,91^. Senolytics dasatinib and quercetin have also succeeded at eliminating senescent cells for patients with diabetic kidney disease^141^. This purports hope for treating LOAD as a neurodegenerative disease of aging, likely advanced by microglial senescence.
